# The innovation value chain of patents: Breakthrough in the patent commercialization trap in Chinese universities

**DOI:** 10.1371/journal.pone.0230805

**Published:** 2020-03-26

**Authors:** Hong Gong, Libing Nie, Yuyao Peng, Shan Peng, Yushan Liu

**Affiliations:** 1 Economics and Management School, Wuhan University, Wuhan, China; 2 Research Center for China Industry-University-Research Institute Collaboration, Wuhan University, Wuhan, China; University of Brescia, ITALY

## Abstract

The innovation value chain is an effective tool for analysing innovation activities and reflects the process of value creation and increase in innovation activities. From the perspective of innovation value chains, we divided patent innovation activities into three stages: knowledge innovation stage, applied research stage and patent commercialization stage. The panel data from 64 universities directly managed by the Ministry of Education from 2009 to 2017 were used and several conclusions were drawn: 1) In the initial stage of knowledge innovation, the fundamental research fund plays a crucial promoting role, and knowledge innovation achievements are mainly published academic papers. 2) In the applied research stage, the knowledge innovation in the early stage and the fund investment in R&D activities have a significant positive effect on the patent output of universities, but the personnel investment has a negative effect. 3) In the final stage of patent commercialization, preliminary research results have a positive impact on patent commercialization, whose marginal effect depends on the industry-university-research relationship, external competition and reputation of the university. The evidence showed that there is a feedback channel between university patent commercialization and knowledge innovation, and new knowledge generated by the interaction with the outside world in the process of patent commercialization was transmitted to the subject of knowledge innovation through this channel, forming a virtuous dynamic cycle. By analysing the driving factors of the value chain of patent innovation in colleges and universities, we provided empirical evidence for the operation mechanism and policy formulation of college patents in China.

## 1 Introduction

Patents are the sources of national scientific and technological innovation. At present, the number of patent applications in China has risen sharply, and China also has the largest number of patent applications in the world. Although the quantity of patent applications in universities has increased dramatically in recent years, the rate of patent commercialization is much lower than what we expected[1]. According to *The State Intellectual Property Office of Patent Statistical Yearbook* (2018), the growth rate of invention patents in colleges and universities in China has accelerated significantly, from 1,548 in 2000 to 19,400 in 2018, an increase of more than 125 times. Nevertheless, the patent transfer rate of universities is only 1.4% (annual patent statistics of the National Intellectual Property Administration, 2018), and most patents are, regrettably, in a "sleep" state. According to statistics of the AUTM licensing activity survey (2018), in 2017, the number of patents disclosed by American universities and research institutes was 24,998, and the number of patent licenses was 7,798, with a licensing disclosure ratio of approximately 31.2%.

However, it is difficult to break through the bottleneck of turning patents from universities into productivity if we only pay attention to a certain process and do not consider it systematically. We started our research from the concept of the innovation chain that was propose by Hage and Hollingsworth in 2000[2]. Through in-depth analysis of its operating mechanism, we identified a value chain of patent innovation in universities. The structure of this paper is the following. Section 2 provides a theoretical framework for the innovation value chain approach used in this contribution. Then, we put forward a research hypothesis based on the three-stage framework and relevant documents. Section 3 gives a brief description of the research methodology. In section 4, empirical results are presented and discussed. Finally, in section 5, we draw conclusions and provide recommendations for each stage.

## 2 Conceptual framework and research hypothesis

### 2.1 Conceptual framework

**Fig 1:**
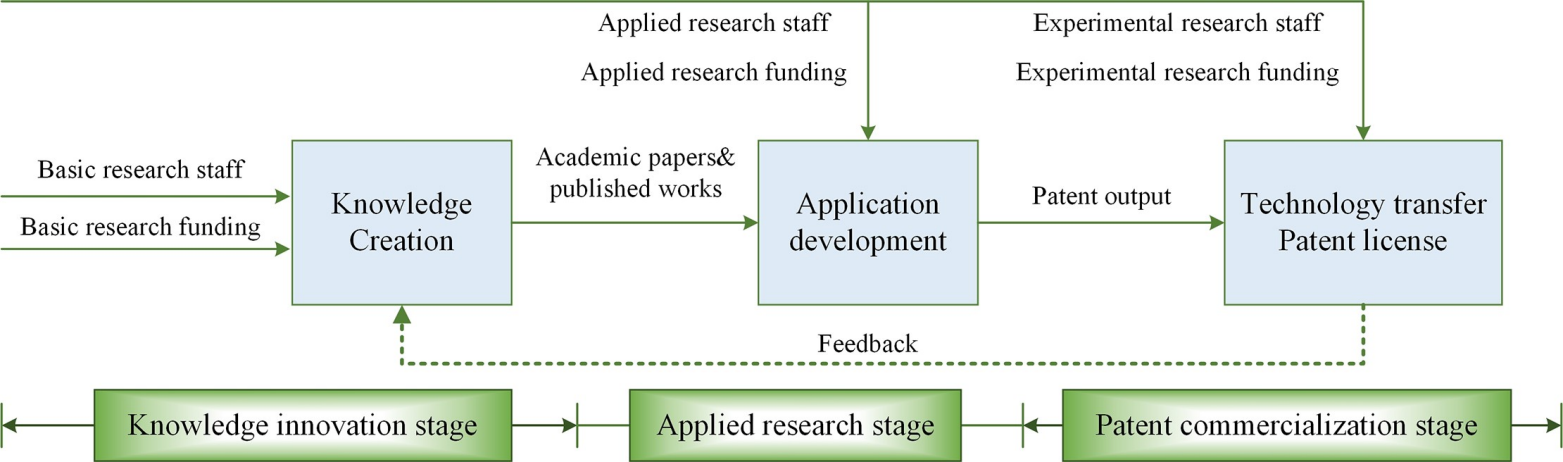
The framework of the patent innovation value chain in universities.

The university patent innovation value chain, UPIVC for short, refers to the process of knowledge innovation, application and commercialization that makes the patent value increase continuously and realizes the amplification of economic benefits. Since Hage and Hollingsworth proposed the concept of the innovation chain in 2000[2], many scholars have been integrating it with practice[3]. Roper et al. (2008) concluded that the innovation value chain was comprised of the recursive process of knowledge sourcing, transformation and exploitation[4]. Hansen and Birkinshaw (2007) offered the innovation value chain as a framework for evaluating innovation performance, which comprised three main phases of innovation: idea generation, conversion, and diffusion[5]. Furthermore, Qin et al. (2017) introduced the innovation value chain into the field of scientific research and divided the process into three stages: knowledge acquisition, technological innovation, and value transformation[6]. Currently, it is considered that there should be a dynamic feedback channel in the innovation process that all innovation-related activities may utilize the input or output of others [7]. In this value chain process, there are not only sequential innovation process links but also interactions and feedbacks between different stages, such as the synergy produced by the interaction between academic activities and industry[8]. According to the process characteristics and output form of patent activities in colleges and universities, we define the UPIVC as the whole process from scientific and technological innovation knowledge to patent value realization (as shown in Fig 1), including three stages: knowledge innovation, applied research and patent commercialization, and a dynamic feedback channel.

First, the knowledge innovation stage includes the input of the original basic research and academic papers and publications. Occupying good academic settings and with abundant R&D resources, universities have become the considerable sources of the science and technology innovation system in China. Second, the transformation from the basic scientific research results to the applied one at universities, including patent application and grant, constitutes the applied research stage. Finally, the high-quality patents are transformed into market-oriented forms at the third stage and the final commercialized products for society can be obtained. In addition, we suggest that there is a feedback channel between the patent commercialization stage and the knowledge innovation stage. The new knowledge generated by the interaction with the industry can be fed back to the original subjective by applying this channel. This provides a virtuous cycle that is conducive to promoting the level of knowledge innovation in universities. In conclusion, the UPIVC contains a complete innovation process from knowledge resources to commercial products, reflecting the process of value-added realization.

Briefly, knowledge innovation refers to the progress from basic resources to original inventions; applied research is the progress from original inventions to patent output, including patent application and grant; patent commercialization is the licensing or transformation of patents; and the feedback channel transfers the previous experience and lessons to the front end of the value chain, forming a benign spiralling patent innovation process.

### 2.2 Research hypothesis

In this section, we discuss the value realization progress of UPIVC, which involves the whole process from original knowledge innovation, applied research to patent commercialization.

#### 2.2.1 Knowledge innovation stage

At this stage, universities carry out research activities represented by basic research by putting in the resources necessary for knowledge innovation. Basic research dominates the academic work of universities. It usually originates from personal research interests and organizational goals, with a focus on theoretical knowledge rather than commercial interests[9]. The core idea is generated by individuals; university researchers are driven by the spark of thought and most basic research is funded by the government and conducted in universities or government-funded research institutions.

The output of this stage is mainly knowledge innovation, such as academic papers, published works, or manuscripts, with the first two as the most simple and applicable forms. Being open and universal, research in universities could bring large rewards to society[10]. As the carriers of knowledge information, their importance is not only reflected in the theoretical basis of scientific research activities but also, more importantly, reflected in solving current and future actual problems.

Scholars have conducted research on the influencing factors of university knowledge innovation output. For example, Bonaccorsi et al. (2006) found that the input of human resources and financial resources significantly improved the output of university research, including university published works and academic papers[11]. In addition, it was found that the basic research funding and human resource input significantly increased the number of SCI papers in universities[12].

Therefore, the assumption is proposed as follows:

**H1a:** The input of basic research staff has a positive effect on university academic papers and published works.
**H1b:** The input of basic research funding has a positive effect on university academic papers and published works.

#### 2.2.2 Applied research stage

The second stage is to transform basic research results into applied results represented by patents. In the stage of knowledge innovation, universities mainly carry out knowledge creation through basic research. Basic research has an open and universal nature, which can generate large returns to the whole society[10]. Although basic research continues to dominate academic research in universities, application research has become increasingly important to universities since the early 1970s. Because the basic research results are usually not directly applicable to commercial applications, they are mainly in the form of academic papers and published works. They belong to the original theoretical innovation knowledge and have certain academic significance, but they do not necessarily have practical value and cannot be directly transformed into productivity. Therefore, in order to make the basic research results suitable for commercialization, further application research and development is essential. When commercialization is the final goal, scientific research in universities is usually only the first step in the long process of technology transfer[13]. That is, in the applied research stage, we conduct application research leading to secondary innovation to determine the possible use of the basic research results and to acquire new knowledge.

The National Science Foundation defines application research as research that acquires knowledge or understanding to meet specific or public needs, and such research is often designed to produce future market value. The main output form of application research is the patent. Scholars have carried out a large number of empirical analyses on the influence factors affecting patent output in universities. First, according to the resource-based theory, the ability of university knowledge innovation directly affects the number of patent applications in universities. As a key evaluation index of university knowledge innovation ability, the number of high-quality papers can reflect the ability of university knowledge creation to a certain extent. Therefore, some scholars believe that the stronger the output capacity of university papers, the stronger the promotion of patent research and development [14, 15]. Second, scholars generally believe that research funding support and human resource input are the second major factors affecting patent output. For example, Hausman et al. (1984) have clearly stated that R&D personnel and funding input is significantly related to patent output[16]. In addition, Fu et al. (2010) found that R&D funding and personnel input can promote the improvement of patent output in universities[17].

Therefore, the assumptions are proposed as follows:

**H2a**: The output of academic papers and published works has a positive effect on patent output in universities.
**H2b**: The input of application research funding and application research staff have a positive effect on patent output in universities.

#### 2.2.3 Patent commercialization stage

The purpose of the commercialization of patents in universities is to transform the results of knowledge creation into productivity [18]. Patent commercialization is one of the key processes for the realization of patent value in universities[1]. The focus is using and integrating existing knowledge and technology to create new application products to serve production activities and life. This process converts the knowledge and patents acquired in the first two stages into economic value. The transformation of patents into productivity can occur through a series of formal and informal channels. Foreign universities choose to set up KTOs or TTOs for patent conversion, while Chinese university patents are implemented in the form of license implementation, patent industrialization, patent transfer, and so on[19]. The main mode of the patent commercialization is in the form of patent sale and transfer, and the number of patent sales contracts is used as the measurement standard.

The patent is licensed or sold to enterprises and institutions, which is the final stage of the realization of the patent value of universities. The number of patents sold by universities is affected by the number of patents granted by universities in the previous stage as the former reflects the quality of the later. The transformation of scientific research into commercial products that meet market needs and is suitable for practical use requires a certain amount of resources and time to make substantial improvements[10]. Even if the research results are commercial, they often need to invest more resources and time to transform them into a form that fully meets the needs of the audience and adapts to industrial production. Therefore, patent commercialization also requires a large amount of experimental development funding support and personnel input support. The knowledge obtained from basic research, application research and practical experience will be substantially transformed into a new product suitable for commercialization and market value.

Therefore, the assumptions are proposed as follows:

**H3a**: The patent output has a positive effect on patent commercialization in universities.
**H3b**: The input of experimental development research funding and experimental development research staff has a positive effect on patent commercialization in universities.

In addition, the difficulty of patent commercialization will be affected by the intensity of the industry-academic collaboration, market competition, and university prestige. It is believed that the network alliance established by the organization is the key to acquiring external resources[20]. For universities, cooperation with industry can provide additional research funding for academic research by opening up new research approaches and providing new insights through joint problem solving [21, 22]. The higher the intensity of the industry-academic collaboration is, the higher the intensity of alliances established between universities and enterprises, making patent commercialization is easier.

According to the market-driven theory, technological innovation is positively related to market demand. Furthermore, market demand and market opportunities will promote the development of university innovation. Some scholars point out that the degree of market competition will affect the commercialization of patents. When faced with a higher level of external competition, patent commercialization is better. Universities generally tend to collaborate with companies that are geographically close to them to facilitate face-to-face communication and guidance. From patent technology owners to technology transferees, there is competition between universities in the region. Under the pressure of competition, universities have the incentive to shorten the time to turn their scientific research results into market-oriented products.

In addition, considering the internal factors of universities, the higher the academic prestige of universities, the more obvious the halo effect of universities, which can make patent commercialization easier. Ambos et al. (2008) also emphasize that the prestige or scientific advantage of universities is a key factor in increasing the possibility of knowledge transfer[23]. According to the Matthew effect, for the same level of research, the research of famous scholars can obtain more citations and feedback to themselves to further enhance their reputation. In the same way, a good academic reputation makes the patent commercialization of universities a virtuous cycle. At the organizational level, university prestige can be seen as a “non-competitive resource” that enables universities to conduct commercial development without affecting the potential of their scientific research.

Therefore, the assumptions are proposed as follows:

**H3c**: The intensity of industry-academy-research cooperation positively moderates the relationship between applied research and commercialization in universities.
**H3d**: The degree of market competition positively moderates the relationship between applied research and commercialization in universities.
**H3e**: The prestige of universities positively moderates the relationship between applied research and commercialization in universities.

#### 2.2.4 Feedback channel

Scholars have also carried out much research on whether the patent commercialization itself can influence the knowledge innovation of universities and cause a dynamic mutual promotion effect. Gulbrandsen and Smeby (2005) found through empirical research that scientists who receive any form of external funding are often more productive than those without external funding[24]. With the increase in industry-academic alliances, interactions with industry have become more common, and researchers can derive significant benefits from this industry-academic relationship [25]. Interactions with industry researchers during the commercialization of patents may trigger new research and foster new collaborations [26], which have a positive effect on subsequent university research. This indicates that cooperation with industry in the patent commercialization stage can play an intermediary role at the organizational level, linking past knowledge innovation with future knowledge innovation.

Therefore, considering that patent commercialization is a creative application of scientific research results, there is a re-innovation of knowledge. According to Kline and Rosenberg (2009), this paper sets a feedback channel between the patent commercialization stage and the knowledge innovation stage[7]. The new knowledge generated by the interaction with the industry in the process of patent commercialization can use this channel to feedback to the stage of original knowledge innovation, so that it can form a dynamic cycle, which is conducive to promoting the level of university knowledge innovation. Sengupta and Ray (2017) believe that patent commercialization can play a mediating effect at the organizational level, linking past knowledge innovation with future knowledge innovation[27]. Therefore, we use the mediating effect to test the feedback effect of university patent commercialization on university knowledge innovation.

Therefore, the assumptions are proposed as follows:

**H4a**: Patent commercialization has a positive effect on knowledge innovation in universities.
**H4b**: Patent commercialization in universities plays a mediating effect in the knowledge innovation at different times.

## 3 Methodology

### 3.1 Data

This study uses data from the *Compilation of Scientific and Technological Statistics in High Schools (2009–2017)* summarized by the Department of Science and Technology in the Ministry of Education. In the compilation, statistics from universities serving as education facilities for the Ministry of Education are listed in detail, while the rest are listed in summary. In accordance with the *Statistical Profile Announcement of University-Affiliated Enterprises* released by the Ministry of Education in 2014, enterprises owned by the former account for 47.94% of the total number of university enterprises in China and have unique advantages in the transformation of scientific and technological achievements, especially patents. Briefly, we chose universities serving as education facilities for the Ministry of Education as our subjects. After universities of art, language and finance, which account for 11, were excluded from the sample, 64 universities were left. Observation values from 2009 to 2017 were selected, and the total sample size was 576.

### 3.2 Variables and measurement

In the stage of knowledge innovation, basic researchers and basic research funds are used as resources, and academic papers and published works are the main outcomes. Academic papers and published works are the most simple, applicable and dominant form to measure the achievements in the stage of knowledge innovation. We take them as the dependent variables of this stage and take basic researchers and basic research funds as the independent variables of this stage.

In the applied research stage, the main resource input is not only the output of the previous stage (i.e., academic papers and published works) but also additional applied researchers and applied research funding, and the main outcomes are patent applications and grants. At this stage, the dependent variables are patent application and grant and the independent variables are academic papers and published works, applied researchers and applied research fund investment.

In the final stage of the commercialization of patents, the main resources are invested in addition to the output of the previous stage (i.e., patent application and grant), as well as additional experimental development personnel and funds, and the output of the results is mainly based on the sale of patents. The transformation of scientific works is one of the key processes of the patent innovation value chain in colleges and universities. Here, we only focus on the transformation of the results represented by the commercialization of patent sales, and the number of patent sales contracts is used as an indicator to measure the output of the patent commercialization stage. At this stage, the dependent variable is the patent sale, and the creative application of scientific research results independent variables are patent application and grant, test development personnel and experimental development funds.

### Regional competition intensity

Being close to high-tech enterprises makes it easier for universities to transfer patent technology, and universities located in higher density areas will face greater competition[28]. This paper measures the intensity of regional competition by dividing the number of regional colleges and universities by the number of regional high-tech enterprises.

### Industry-university-research intensity

The resources that universities obtain from the outside can, to a certain extent, represent the strength of the innovation network alliance established by universities[29]. In this paper, the proportion of appropriation of enterprises and institutions in the total appropriation is used to measure the intensity of industry-university-research.

### University prestige

In order to establish world-class universities and high-level universities, some high schools, such as Peking University and Tsinghua University, were listed as key supported project by the Ministry of Education in China. It was named "Project 985". In contrast, universities of "Project 985" often have a valuable social reputation and can obtain a wider range of social resources and government policy support. This paper selects whether universities belong to "Project 985" as the proxy variable of university reputation. The value of universities belonging to "Project 985" is 1, and the value of the rest of universities belonging to "Project 211" is 0.

We take the degree of regional competition, the intensity of industry-university-research cooperation and the reputation of universities as moderating variables and test their moderating effects on the link between patent research and commercialization of universities (see Table 1).

**Table 1:** Variables.

Variables	Definitions	Descriptive Statistics
**Knowledge innovation stage**		
Academic papers	Original scientific research results published in academic journals	Mean:3834.531 SD:3070.189
Published works	Theoretical essays or monographs on scientific issues published by the official publishing department, as well as textbooks and science books	Mean:30.840 SD:22.359
Basic research staff	Annual number of staff engaged in fundamental research	Mean:411.033 SD:475.549
Basic research funding	Annual funding spent in fundamental research	Mean:15.947 SD:20.968
**Applied research stage**		
Patent application	Annual number of patent applications	Mean:625.681 SD:647.314
Patent grant	Annual number of patents granted	Mean:374.496 SD:427.226
Application research staff	Annual number of staff engaged in application research	Mean:415.005 SD:381.112
Application research funding	Annual funding spent in application research	Mean:21.507 SD:24.314
**Patent commercialization stage**		
Patent sales	Annual number of patent sales contracts	Mean:13.5 SD:27.317
Experimental staff	Annual number of staff engaged in experimental development research	Mean:107.773 SD:146.965
Experimental funding	Annual funding spent in experimental development research	Mean:7.008 SD:11.309
Intensity of industry-academic collaboration	Proportion of the funding allocated by enterprises and institutions on the total funding	Mean:0.342 SD:0.201
Degree of market competition	Number of high-tech enterprises divided by the number of higher education institutions	Mean:0.129 SD:0.0923
University prestige	Assign 1 when the university belongs to the 985 project and 0 otherwise	Mean:0.516 SD:0.500
Total research staff	Total number of staff engaged in R&D activities	Mean:840.283 SD:756.554
Total research funding	Total funding spent in R&D activities	Mean:44.462 SD:44.176

### 3.3 Model

To test hypotheses 1a-3b, the regression model is constructed as follows:

$$Y_{i,t}=\beta_{0}+\beta_{1}X_{i,t-1}+\beta_{2}{Fund}_{i,t}+\beta_{3}{Labor}_{i,t}+\varepsilon_{i,t}$$ (1)

where *Y_i,t_* is the output of the university (including the output of university academic papers and published works, and the patent output of universities), *X*_*i,t*−1_ is the output of the university in the last year, *Fund_i,t_* is the funding input of the university, and *Labor_i,t_* is the staff input of the university.

To test the moderating role of the intensity of industry-academic collaboration, the degree of market competition and the university prestige, the regression model is constructed as follows:

$${Commercialization}_{i,t}=\beta_{0}+\beta_{1}{Patent}_{i,t-1}+\beta_{2}X_{i,t}+\gamma Z_{i,t}+\varepsilon_{i,t}$$ (2)

where *Commercialization_i,t_* stands for patent sales of the university, *Patent*_*i,t*−1_ is the number of patent applications of the university, *X_i,t_* is the interaction term with the intensity of industry-academic collaboration, the degree of market competition and the university prestige, and *Z_i,t_* are control variables.

To control the heterogeneity of unobservable individuals in the random effect model and control the data loss in the construction process, we used the number of patent sales in the last three years to create the pre-sample value of the dependent variable, namely, the average number of patent sales t-1 to t-3. The pre-sample estimator is then used as an additional control variable for regression.

Furthermore, to test whether the patent commercialization of the previous stage can be used as a mediator variable to feedback university knowledge innovation in next stage, the regression model is constructed as follows[27]:

$${Knowledge}_{i,t}=\beta_{0}+\beta_{1}{Knowledge}_{i,t-1}+\beta_{2}{Commercialization}_{i,t-1}+\gamma Z_{i,t}+\varepsilon_{i,t}$$ (3)

where *Knowledge_i,t_* is the number of academic papers from universities.

## 4 Results

### 4.1 Realization process in UPIVC

Table 2 demonstrates the process of the knowledge innovation stage and the applied research stage in Chinese universities. After the Hausmann test, the fixed effect model should be used theoretically. However, there were too many zeros in the patent sales, which may lead to excessiveness identification. In addition, there were also variables that did not change with time, so adopting a negative binomial random effects model was more prudent. The research results showed that in the stage of knowledge innovation, the investment of basic research funding was of benefit to the output of academic papers in universities, but the impact of the input of basic research staff was not significant. The personnel input in the statistics includes not only those who directly participate in the basic research project activities but also the managers and service personnel who provide information service, materials supply, equipment maintenance and other services. In addition, it is not significant when using published works as the dependent variable. The scientific published works from universities do include theoretical papers or monographs on scientific and technical issues, the majority of which, however, are textbooks. These achievements are derived from the daily academic or teaching experience, not experimental evidence or theoretical considerations. In the applied research stage, the results in Table 2 showed that the output of the academic papers in the knowledge innovation stage significantly contributes to the patent output in universities. This is because the university's patent output is related to the university's ability to create new knowledge, which can be reflected in the number of published papers to a certain extent. Moreover, the input of application research funding had a significant positive effect on the patent output in universities. Notably, the input of staff had a negative effect on the patent output, counter intuitively, indicating that the personnel in the application research are valued for their quality, not their number. In conclusion, the paper proved that the past knowledge innovation had indeed strengthened the follow-up applied research.

**Table 2:** Knowledge innovation stage and applied research stage.

	Academic papers	Published works	Patent application	Patent grant
	(1)	(2)	(3)	(4)
Basic research funding	0.00345*** (3.77)	-0.00282* (-1.73)		
Basic research staff	0.0000799 (1.09)	0.000123 (1.39)		
Application research funding			0.00864*** (6.82)	0.00951*** (6.79)
Application research staff			-0.000581*** (-4.06)	-0.000548*** (-3.39)
Academic papers T-1			0.000112*** (8.07)	0.000141*** (9.23)
Published works T-1			0.000365 (0.29)	-0.00126 (-0.89)
Time fixed effect	Yes	Yes	Yes	Yes
Individual fixed effect	Yes	Yes	Yes	Yes
Cons	2.536*** (37.34)	1.908*** (20.09)	1.411*** (14.76)	0.928*** (9.58)
N	576	576	576	576
Wald chi-square	25.1***	3.7	129.8***	174.6***
Log likelihood	-4037.915	-1872.358	-2833.370	-2661.762

t Statistics in parentheses

*p<0.05

**p<0.01

***p<0. 001.

To verify the hypothesis in the patent commercialization stage, we used a negative binomial random effects model in consideration of excessiveness identification. It is reasonable that patent output is positively related to patent commercialization in universities, as the former is the basis of the latter (see Table 3). At the same time, we found that the degree of external competition and the intensity of the industry-academic collaboration played positive roles in moderating the relationship between patent grants and future patent commercialization activities, while university prestige was in contrast. Under the pressure of competition, universities have more incentives to shorten the time to convert research into market-oriented products. External resources from the industry-academic alliance may also be directly invested in market-oriented research and development. With the increase in competition and the industry-academic alliance, the positive marginal effect of patent grants had been significantly enhanced in the past, accelerating the process of patent commercialization in universities.

**Table 3:** Patent commercialization stage.

	Dependent variables: Patent sales			
	(1)	(2)	(3)	(4)
Patent grant T-1	0.000402*** (3.62)	0.000786*** (4.87)	0.000252** (2.15)	0.000406*** (3.85)
Experimental development funding	0.00371 (0.65)			
Experimental development staff	0.000518 (1.22)			
Degree of competition		-0.594 (-0.74)		
Degree of competition* Patent grant T-1		0.00646*** (3.77)		
Intensity of industry-academic collaboration			-0.109 (-0.30)	
Intensity of industry-academic collaboration* Patent grant T-1			0.00266** (3.58)	
Prestige				0.345** (2.20)
Prestige *Patent grant T-1				-0.000824*** (-4.19)
Average number of patent sales in the last 3 years	0.00949*** (3.42)	0.00899*** (3.06)	0.0108*** (3.78)	0.0100*** (3.49)
Time fixed effect	Yes	Yes	Yes	Yes
Individual fixed effect	Yes	Yes	Yes	Yes
Cons	-0.0301 (-0.23)	0.0442 (0.27)	0.183 (0.99)	-0.0858 (-0.57)
N	576	576	576	576
Wald chi-square	33.7***	45.0***	45.3***	59.6***
Log likelihood	-1620.724	-1614.974	-1616.919	-1613.521

t Statistics in parentheses

*p<0.05

**p<0.01

***p<0.001.

However, we did not find clear evidence to support H3e. The positive marginal effect of the patent development in the past on patent commercialization was obviously weakened as the popularity of universities generally increased. We speculated that this may be because the knowledge generation and knowledge share was path dependent within the university. The higher the academic prestige of universities, the easier it is to form a virtuous cycle in the applied and experimental development research activities and achieve self-sufficiency to some level. The Matthew effect shows that prestigious scholars have more citations and feedback to further enhance their reputation. Therefore, we believe that university prestige causes the internal path dependence effect of the two activities to gradually increase, which significantly weakens the positive marginal effect of the patent grant on patent commercialization.

### 4.2 Feedback channel in the innovation value chain of patents

Table 4 analyses the feedback role of university patent commercialization on knowledge innovation. After Hausmann's test, the negative binomial fixed-effects regression model was applied. There were only control variables in column (1), and knowledge innovation in the past stage was added to column (2). The coefficient was significantly positive, which indicates that there exists a path-dependent effect in the knowledge innovation stage. Column (3) introduced the patent sales of the last three years as the mediator variable, and its coefficient was also significantly positive, signifying that patent commercialization can be fed back to the next knowledge innovation stage through the mediation effect. Thus, H4a and H4b were verified in a careful way. In addition, Table 4 shows that the total staff in R&D does not contribute to the outcome of the knowledge innovation. Only the funding positively influences the number of academic papers, which reflects the possibility of a scale effect in the investment of university knowledge innovation.

**Table 4:** The feedback of patent commercialization on knowledge innovation.

	Dependent variables: Academic papers		
	(1)	(2)	(3)
Academic papers T-1		0.000109*** (12.44)	0.000108*** (12.33)
Patent sales T-1			0.00107* (1.80)
Total research staff	0.0000367 (0.80)	-0.0000208 (-0.46)	-0.0000202 (-0.45)
Total research funding	0.00230*** (4.78)	0.000911** (2.17)	0.00103*** (2.46)
Time fixed effect	Yes	Yes	Yes
Individual fixed effect	Yes	Yes	Yes
Cons	2.503*** (34.87)	2.557*** (32.57)	2.550*** (32.39)
N	576	576	576
Wald chi-square	33.0**	199.3***	206.9***
Log likelihood	-4034.736	-3455.143	-3453.602

t Statistics in parentheses

*p<0.05

**p<0.01

***p<0. 001.

We must consider strengthening the role of basic scientific research if we aim to improve the patent commercialization of universities. A patent commercialization activity that could be fed back to knowledge innovation helps to create a virtuous circle. Universities that focus on basic scientific research could have a higher level of knowledge transfer, some of which can further enhance its knowledge innovation through commercialization, as interaction with industries will stimulate some new, relevant, practical and even bold ideas. The income from patent commercialization may also be invested in knowledge innovation, enhancing the ability of scientific research and having a positive effect on it in the future. In addition, from Table 4, the coefficient of patent sales is much larger than that of knowledge innovation, which indicates that the indirect mediation effect of patent commercialization is larger than the independent effect in knowledge innovation activities.

## 5 Conclusion

There is a non-linear innovation value chain in the patent activities of colleges and universities, involving the whole process of value realization from knowledge innovation and applied research to patent commercialization. In the initial stage of knowledge innovation, basic research funding played a crucial promoting role, and knowledge innovation results were presented in the form of published academic papers. In the applied research stage, the early knowledge innovation and funding investment in R&D activities had a significant positive impact on patent output, but personnel input triggered a significant negative impact. Therefore, in regard to applied research, universities need to pay special attention to the construction of research teams. In the final stage of patent commercialization, early research achievements, serving in a dominant role, had a significant positive impact on later patent commercialization, which depends on the external competition faced by universities, the establishment of the industry-university-research relationship and their own reputation. When universities were confronted with intense competition and relationship, there would be a pressing need for patent transformation. For the academic universities with high prestige, however, due to the inherent inertia of scientific research, they may demand relatively more external input in terms of patent commercialization. In addition, we found that there was a feedback channel between patent commercialization and knowledge innovation. The new knowledge generated from the interaction with industry in the process of patent commercialization could be fed back to the main body of knowledge original innovation through this channel to increase the level of knowledge innovation.

Although the contradiction between scientific research and patent commercialization still exists on a personal level, researchers engaged in commercial activities may postpone their publication of works or even choose not to disclose them. However, universities may not need to over-focus on short-term or medium-term research. Under the path of patent commercialization driven by intellectual property, there is a virtuous circle in the UPIVC. In accordance with it, we suggest universities allocate more resources at the stage of knowledge innovation when establishing the innovation value chain of patents. Once a good research foundation is formed and the feedback channel is constructed, commercialization could play a key role in strengthening knowledge innovation.

Our research does not come without limitations. Firstly, in the sample selection stage, we selected carefully and focused more on representative scientific and technological achievements to realize the goal of analysing the whole value chain of knowledge innovation, applied research and commercialization in universities. It inevitably raises concerns about the universality of our results. We expect that our research could be applied to the analysis of patent activities in universities with whole value chains at the same time. But if universities are only active in basic research or, on the contrary, on applied research, the results may show quite different characteristics. Second, selling patents is just one of the ways through which knowledge and technology in universities can be transferred to enterprises. The ways of patent exploitation also include patent license, independent implementation and patent industrialization. Although these activities need different forms of management when they are profitable, it is meaningful to take them as the indicators of early patent transformation. In the future, a direction of our research is to consider expanding the index range to analyse the patent transformation in universities more comprehensively. Third, the investment of additional trial developers and trial development funds in the stage of patent commercialization is uncontrollable.

## Supporting information

S1 Data (XLS)
